# Meeting the needs of people with physical disabilities in crisis settings

**DOI:** 10.2471/BLT.19.246918

**Published:** 2019-12-01

**Authors:** Cornelia Anne Barth

**Affiliations:** a International Committee of the Red Cross, 19, avenue de la Paix 1202, Geneva, Switzerland.

An estimated 20 million of the 135 million people who need humanitarian assistance globally are living with some form of disability and require access to rehabilitation services and assistive technology.^1^ This figure excludes the unknown number of people with conflict-caused impairment. However, physical disabilities and rehabilitation needs of populations in conflict-affected settings are rarely considered and very few international organizations specializing in rehabilitation are active in such contexts.

While two important World Health Organization (WHO) initiatives on rehabilitation were launched in the past years, they do not fully capture the challenges of protracted crises. One such action was the Emergency Medical Teams Initiative, focusing on sudden onset disasters, which differ in nature and response from protracted crises.^2^ The second initiative was Rehab 2030, which aims to advance the global rehabilitation agenda around the health-related sustainable development goal (SDG) 3.^3^ This initiative targets low- and middle-income countries that request assistance in anchoring rehabilitation in their health system. However, the initiative starts at a level of systems organization that does not exist in most countries of protracted crisis.

Rehabilitation for persons with permanent impairment and disability is demanding in any given context, because it involves long-term interventions and requires financial resources and care-providers with specialized skills. In protracted crises, however, rehabilitation is particularly challenging.

The overall situation may be characterized by political uncertainty, poverty and corruption. The infrastructure may be destroyed or degraded, affecting communication, access to buildings and roads, and procurement of rehabilitation products. The security situation may be volatile and unpredictable. The education system may be weak, complicating professional training. The health system may be overwhelmed or dysfunctional. The needs for rehabilitation will be increased, urgent and complex. Therefore, clinical presentations requiring rehabilitation will be pre-existing conditions that were deteriorating or further developing, plus multiple physical and psychological trauma accumulated at different points in time. Cultural concepts and beliefs around disability may be stigmatizing, hindering the development of rehabilitation policies. Accordingly, the most vulnerable, including women and children with disabilities, may get further marginalized, exploited and exposed to violence. Rehabilitation professionals, if existent, may have left the country, or been killed in conflict,^4^ and there may not be sufficient numbers of qualified remaining staff.

Given this complexity and magnitude of needs, specific approaches are required.

First, greater awareness of the rehabilitation needs in countries of protracted crisis is needed. Raising awareness is done best by including persons concerned and their rehabilitation providers. In 2019, the International Committee of the Red Cross (ICRC) sponsored over 50 individuals from 26 countries to participate in the World Confederation for Physical Therapy congress^5^ to promote exchange between professionals from high-income and protracted crisis settings. Other ways to raise awareness are publications in the media reaching beyond the rehabilitation or aid community. 

Second, rehabilitation programmes should be designed and managed to get users ready for social reintegration. Organizations active in these settings could make their employment system as inclusive as possible and proactively offer perspectives for persons with disabilities, by engaging in team diversity and ensuring accessibility for persons with various forms of disabilities to allow them to advance in their career paths.

Third, sustainability should be strengthened by capacity building of staff, through advocacy with relevant civil society organizations and authorities, and the creation of structures in the absence of a state system. Moreover, employing former rehabilitation users with disabilities in rehabilitation structures contributes to sustainability.

Fourth, guidelines on clinical work in challenging settings, such as those by the World Confederation for Physical Therapy, the ICRC and partner organizations are crucial.^6^^–^^8^ These guidelines should eventually expand their scope on all aspects of functionality including participation and reintegration. The same applies to WHO’s Blue Book^9^ on sudden onset disasters and the Red Book, currently being developed and specifically designed for conflict response. 

Fifth, mainstreaming rehabilitation in protracted crises requires close collaboration with international rehabilitation or medical associations and WHO, especially on the use and context-adaptation of documents developed within the Rehab 2030 initiative. In crisis settings, collaborating with aid organizations to systematically raise awareness on rehabilitation needs, is vital.

Sixth, data and research are needed on demographics and clinical presentations, on the impact of rehabilitation interventions and on workforce. Equally important is qualitative, participatory research that includes the views and needs of affected populations.

If the strategies above are considered, health for all as the priority towards achieving SDG 3 can also be accomplished with regards to rehabilitation needs of people in protracted crises.
